# S-band hybrid amplifiers based on hydrogenated diamond FETs

**DOI:** 10.1038/s41598-020-75832-w

**Published:** 2020-11-04

**Authors:** Walter Ciccognani, Sergio Colangeli, Claudio Verona, Fabio Di Pietrantonio, Domenico Cannatà, Massimiliano Benetti, Vittorio Camarchia, Marco Pirola, Patrick E. Longhi, Gianluca Verona Rinati, Marco Marinelli, Ernesto Limiti

**Affiliations:** 1 Electronics Engineering Department, Università di Roma Tor Vergata, 00133 Rome, Italy; 2 Industrial Engineering Department, Università di Roma Tor Vergata, 00133 Rome, Italy; 3 Istituto di Acustica e Sensoristica, Orso Mario Corbino”, 00133 Rome, Italy; 4 Electronics and Telecommunications Department, Politecnico di Torino, 10129 Turin, Italy

**Keywords:** Electrical and electronic engineering, Electronic devices

## Abstract

The first realizations of S-band hybrid amplifiers based on hydrogenated-diamond (H-diamond) FETs are reported. As test vehicles of the adopted H-diamond technology at microwave frequencies, two designs are proposed: one, oriented to low-noise amplification, the other, oriented to high-power operation. The two amplifying stages are so devised as to be cascaded into a two-stage amplifier. The activities performed, from the technological steps to characterization, modelling, design and realization are illustrated. Measured performance demonstrates, for the low-noise stage, a noise figure between 7 and 8 dB in the 2–2.5 GHz bandwidth, associated with a transducer gain between 5 and 8 dB. The OIP3 at 2 GHz is 21 dBm. As to the power-oriented stage, its transducer gain is 5–6 dB in the 2–2.5 GHz bandwidth. The 1-dB output compression point at 2 GHz is 20 dBm whereas the OIP3 is 33 dBm. Cascading the measured S-parameters of the two stages yields a transducer gain of 15 ± 1.2 dB in the 2–3 GHz bandwidth.

## Introduction

Within the frame of space-oriented technologies, a growing need is being experienced for amplifiers capable of handling large power densities at high frequencies, therefore exploiting wide-bandgap semiconductors^1,2^. Indeed, whereas vacuum devices are still the only viable options for certain satellite applications^2^, continuous technological innovations are leading to gradually replacing them with increasingly smaller and more light-weight solid-state transistors, starting from the lower portions of the spectrum.

Diamond is featured, as a material, by exceptional physical, mechanical and chemical properties, such as high carrier mobility (2100 cm^2^ V^−1^ s^−1^), low relative dielectric constant (5.7), wide energy gap (5.5 eV) and superior thermal conductivity (up to 2400 W m^−1^ K^−1^)^3–8^. In particular, diamond features nearly double maximum electric field and more than ten times the thermal conductivity of GaN, which is the foremost semiconductor for state-of-the-art high-power applications at microwave frequencies ^6^. In these terms, diamond decidedly outperforms all other materials currently used to fabricate electronic devices (Si, GaAs and GaN) and is deemed, as a consequence, to be the likeliest successor of GaN in high-frequency applications requiring large power densities and survivability to significant fluxes of ionizing radiation ^9^. These include space-borne applications as well as others in hostile environments (e.g., nuclear reactor monitoring and radiotherapy dosimetry). Diamond transistors can be expected to deliver RF power in excess of 200 W at a few GHz, after its technology readiness level (TRL) has reached an acceptable position, therefore surpassing the performance achieved by commercially available GaN transistors^10,11^.

Nevertheless, several technological problems, such as reproducibility and performance stability over time^12–18^, still hinder from fully exploiting the potential of diamond. Moreover, notwithstanding numerous demonstrations of active processes on diamond from different research groups worldwide, the TRL is quite low, between 2 and 3, due to the lack of many technological steps necessary to reach industry-level production. Finally, very few attention has been given so far to the modelling of diamond-based transistors as a fundamental step towards the design and realization of the first microwave amplifiers in this technology.

To move the first steps towards a higher TRL, a research project was specifically proposed by the Authors, entitled “Reliable Microwave devices on hydrogenated Diamond for space Applications” (ReMiDA) and financed by the Italian Space Agency (ASI). The main objective is the first demonstration of microwave hybrid amplifiers based on diamond transistors.

## Methods

### Technology

High-quality, single-crystal, 1 µm-thick diamond films were grown by Plasma-Enhanced MicroWave Chemical Vapor Deposition (PE-MWCVD) technique on commercial, low-cost, synthetic diamond substrates 4.5 × 4.5 × 0.5 mm^3^ in size and (100)-oriented. The diamond films were treated with hydrogen plasma to terminate the surface with hydrogen bonds, which makes it conductive. More in detail, the following procedure was followed: first, interrupt CH_4_ flux, expose the sample to hydrogen plasma for 30′ with an increased H_2_ flux of 120 sccm and pressure of 150 mbar.

**Figure 1:**
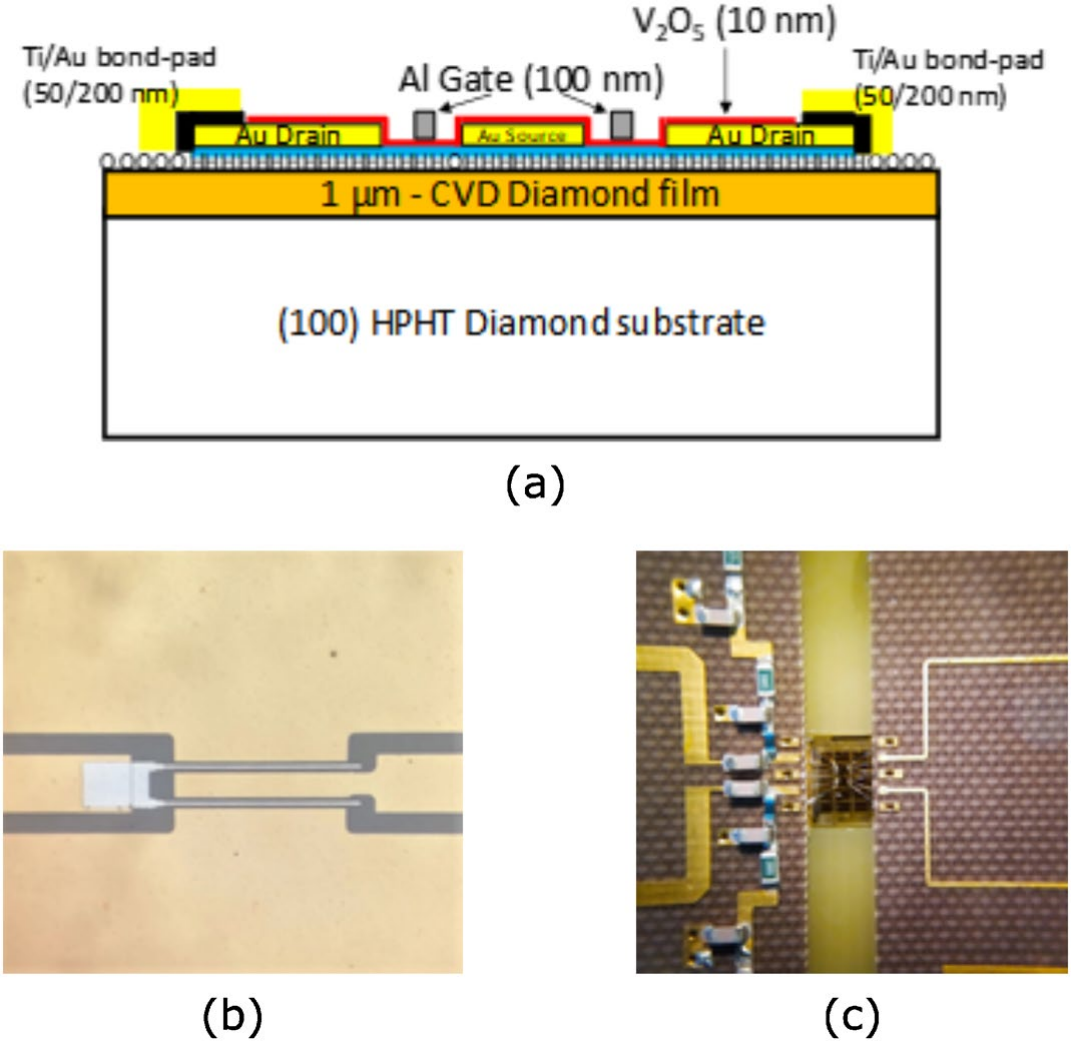
(**a**) Schematic cross section of the diamond MISFETs. (**b**) Detail of the MISFET active region. (**c**) Detail of the microwire bonding between the HPA passive networks and the transistor.

The MISFETs were fabricated on a hydrogenated CVD diamond layer. They have a coplanar layout with two gate fingers of 1 µm length, 75 and 150 µm widths. The device fabrication process consists in the following steps. A gold film, 200 nm in thickness, was thermally evaporated on hydrogenated diamond surface to form the ohmic contact on it. The Au mask, patterned by standard photo-lithographic techniques, was used as the electrode for the source and drain contacts. In order to electrically insulate the devices on the same diamond substrate, the surface was oxidized by Reactive Ion Etching (RIE) in O_2_ gas. A second photolithography was performed to define the bilayer Ti/Au (50 nm/200 nm) bond-pad structures used to wire bonding the devices. Then, a third photolithography and Au wet etching by KI/I_2_ were performed to define a source-to-drain gap of about 3.5 μm. Subsequently, a thickness of about 10 nm of V_2_O_5_ was thermally evaporated on the whole active diamond surface after an annealing treatment at 250 °C in vacuum^14^. Finally, a 1 µm-long gate was fabricated by thermal evaporation of aluminum (100 nm in thickness) and lift-off technique. Channel geometry was chosen on the basis of previous studies^19^. The oxide and metal layer thicknesses were measured by a calibrated quartz microbalance during the evaporation technique. A schematic cross-sectional view of diamond MISFETs and an optical photo of the device active region are shown in Fig. 1(a) and (b), respectively. Finally, the diamond substrate was glued on a dedicated PCB (as detailed in the section about design) and aluminum wire (25-μm in diameter and about 2.5-mm long) was used to ultrasonic micro-bond diamond devices to external circuits, as clearly seen in Fig. 1(c).

### Characterization and modelling

The characterization campaign included DC, small-signal, noise figure, load pull and compression measurements. Drain and gate voltages were limited to negative values down to − 20 V and − 4 V, respectively, to safeguard the devices from degradation, which occurs as a gradual, irreversible shrink of the output I-V curves. Also, DC power and current densities were limited as follows: *P*_*D*_/*W* ≤ 3 W/mm and *I*_*D*_/*W* ≥ − 350 mA/mm. These limitations are the result of the experience gained with this technology in the past^13^ and in recent work carried out by some of the authors^20^. For an explanation on the degradation mechanisms in H-diamond devices, the Reader is referred to^17^.

S-parameters were measured up to 40 GHz by means of a custom Thru-Reflect-Line calibration kit realized on a diamond substrate identical to those carrying the active devices. The kit comprises four “line” standards (THRU, LINE1 to LINE3), ranging from 460 to 3260 µm, and two “reflect” standards (OPEN and SHORT). The track and gap width of the CPW lines are 8 µm and 36 µm, respectively. Clearly, the access geometry is the same as for the active devices to be measured. As a reference, the attenuation constant of the lines range from 0.5  to 0.75 dB/mm for frequencies between 1.5 and 6 GHz.

The best values of cutoff frequency (*f*_*T*_) and maximum frequency of oscillation (*f*_*max*_) found at the device reference planes are 4.18 GHz and 16 GHz, respectively.

Matched-load noise figure (*NF*_50_) was measured in the 5–15 GHz band with the Y-factor technique, by exploiting a solid-state noise source with nominal excess-noise ratio (ENR) of 5 dB. Typical *NF*_50_ values in the measurement band range from 11 to 15 dB, depending on frequency and operating point: so high values are a consequence of the remarkable distance of the optimum noise reflectance (Γ_*opt*_) from the origin of the Smith chart. Incidentally, similar considerations hold true for the reflectances for simultaneous conjugate match and optimum output power.

**Figure 2:**
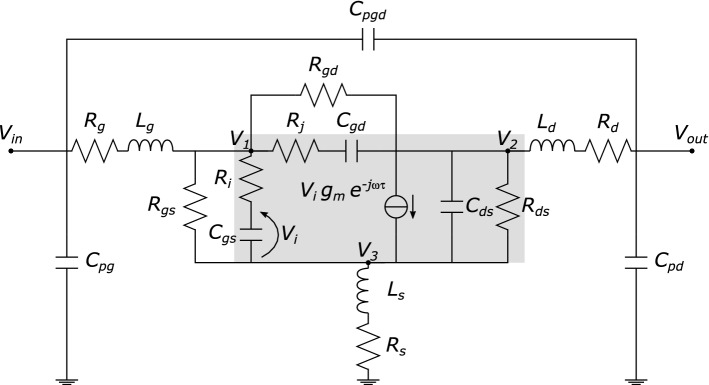
Equivalent circuit adopted for the small-signal and noise modelling of the diamond MISFETs.

Based on small-signal measurements of the whole 2× family realized (unit gate peripheries of 25 µm, 50 µm, 75 µm, 100 µm and 150 µm), a scalable equivalent-circuit model was also extracted by means of the approach in^21^, whose schematic is reported in Fig. 2. Normalized transconductance *g*_*m*_/*W* was found to be approximately 120 mS/mm, whereas the channel time delay τ was about 7 ps. The extrinsic resistors were found to be nonnegligible: *R*_*G*_ ≈ 7 Ω, *R*_*S*_∙*W* ≈ *R*_*D*_∙*W* ≈ 9 Ω∙mm. The scalable model was also equipped with equivalent noise temperatures, extracted from *NF*_50_ measurements as explained in^21^. As from previous experience with the technology, the optimum terminations for available gain and noise figure are far from the standard 50 Ω load: this entails practical difficulties when matching for gain, noise or the (best) trade-off of the two^22^, because the external reference impedances are clearly out the designer’s control.

Large-signal characterization was carried out on devices with periphery 2 × 150 µm at 2.5 GHz. In particular, load-pull measurements were performed to locate the optimum load impedance for output power, which resulted to be *Z*_*L*_ = (260 + j90) Ω at 2 GHz. The maximum output power resulted to be 17 dBm at 5-dB compression, associated with a 23% efficiency.

### Amplifier design approach

Based on the figures of merit obtained for the realized transistors, the goal was set of designing a two-stage S-band hybrid amplifier, featured by at least 10 dB of gain. However, as a measure to minimize risk and explore different kinds of design, the two stages were designed and realized as independent amplifiers, one oriented to low-noise operation (LNA), the other to high power (HPA).

The designs were carried out based on a low-loss, low-dielectric constant substrate: the latter feature was selected to reduce sensitivity to tolerances in PCB realization. In particular, a Diclad-870 substrate was used, featured by a height of 760 µm and a relative dielectric constant of 2.33 and a loss tangent of 0.0009. In addition to the amplifying stages, a custom Line-Reflect-Reflect-Match (LRRM) cal kit was designed: the amplifiers and cal kit are compatible with Ground-Signal-Ground RF probes with 750 µm pitch. The input and output matching networks (IMN and OMN, respectively) of the LNA and HPA also include RF-DC decoupling subnetworks and DC feeds, the latter accessible with single-finger probes.

**Figure 3:**
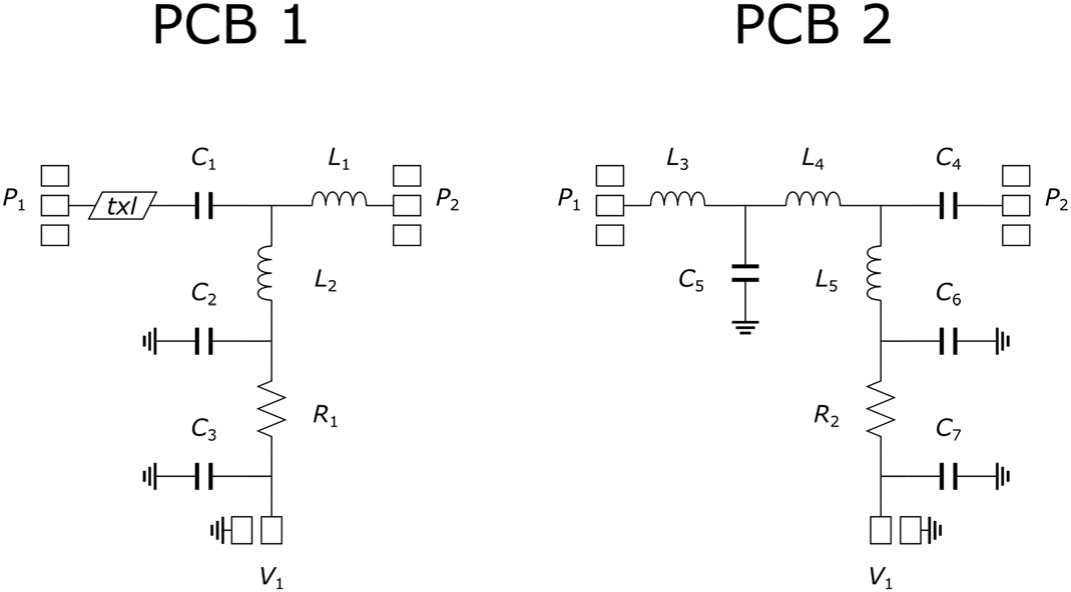
Schematics of the two cards implementing the matching networks of the LNA stage. Input and output RF pads are denoted as *P*_1_ and *P*_2_, respectively. DC pads are denoted as *V*_1_.

**Figure 4:**
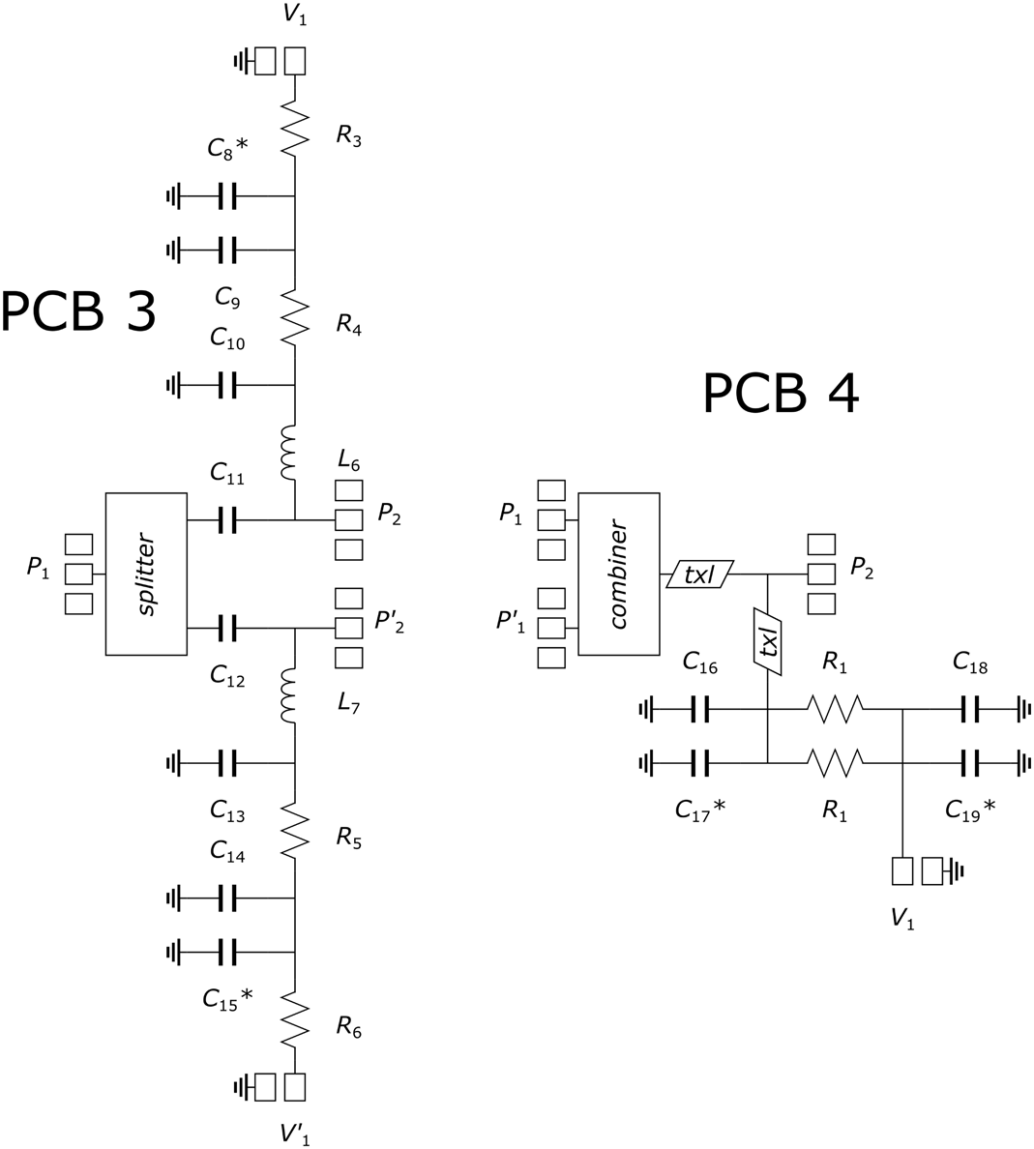
Schematics of the two cards implementing the matching networks of the HPA stage. Input and output RF pads are denoted as *P*_1_ (or *P*′_1_) and *P*_2_ (or *P*′_2_), respectively. DC pads are denoted as *V*_1_ (or *V*′_1_).

The five cards corresponding to the four matching networks and to the cal kit were arranged into a single panel, subsequently cut in the necessary pieces. Then, the PCBs were completed by soldering surface-mount devices (SMDs) with standard (imperial) sizes of 0402, 0603 and 1206. The schematics of the four cards implementing the matching networks of the LNA and HPA stages are reported in Figs. 3 and 4, respectively. Labels denoted with asterisks indicate that the component is optional. The component details are listed in Table 1.

**Table 1:** List of components of the matching networks.

Component label	Part number	Manufacturer	Nominal value
R1, R2, R4, R5	TNPW0603 10R0 BEEN	Vishay	10 Ω
R3, R6	TNPW0603 4K70 BEEN	Vishay	4.7 kΩ
R7, R8	CRCW120610R0FKEAC	Vishay	10 Ω
L1	994-0402DC-2N4XJRW	Coilcraft	2.4 nH
L2	994-0402DC-2N8XJRW	Coilcraft	2.8 nH
L3	994-0402DC-16NXGRW	Coilcraft	16 nH
L4	994-0402DC-6N2XJRW	Coilcraft	6.2 nH
L5	994-0402DC-3N9XJRW	Coilcraft	3.9 nH
L6, L7	994-0402DC-4N7XJRW	Coilcraft	4.7 nH
C1	CBR04C708B5GAC	Kemet	0.7 pF
C2, C3, C6, C7	CBR04C100J5GAC	Kemet	10 pF
C4	CBR04C109B5GAC	Kemet	1 pF
C5	CBR04C208B5GAC	Kemet	0.2 pF
C8*, C9, C10, C13, C14, C15*, C16, C17*, C18*, C19*	CBR06C359B5GAC	Kemet	3.5 pF
C11, C12	CBR06C169B5GAC	Kemet	1.6 pF

Based on a LRRM calibration, passive networks were ensured to behave according to simulations. Similarly, the active devices on the diamond sample were screened to discard those not working: this step is not critical since just one transistor is needed for the LNA stage and two for the HPA, and a whole 5 × 4 matrix of the desired periphery is available for either stage. However, the two HPA devices are required to lie on the same column to allow minimum-length bonding.

**Figure 5:**
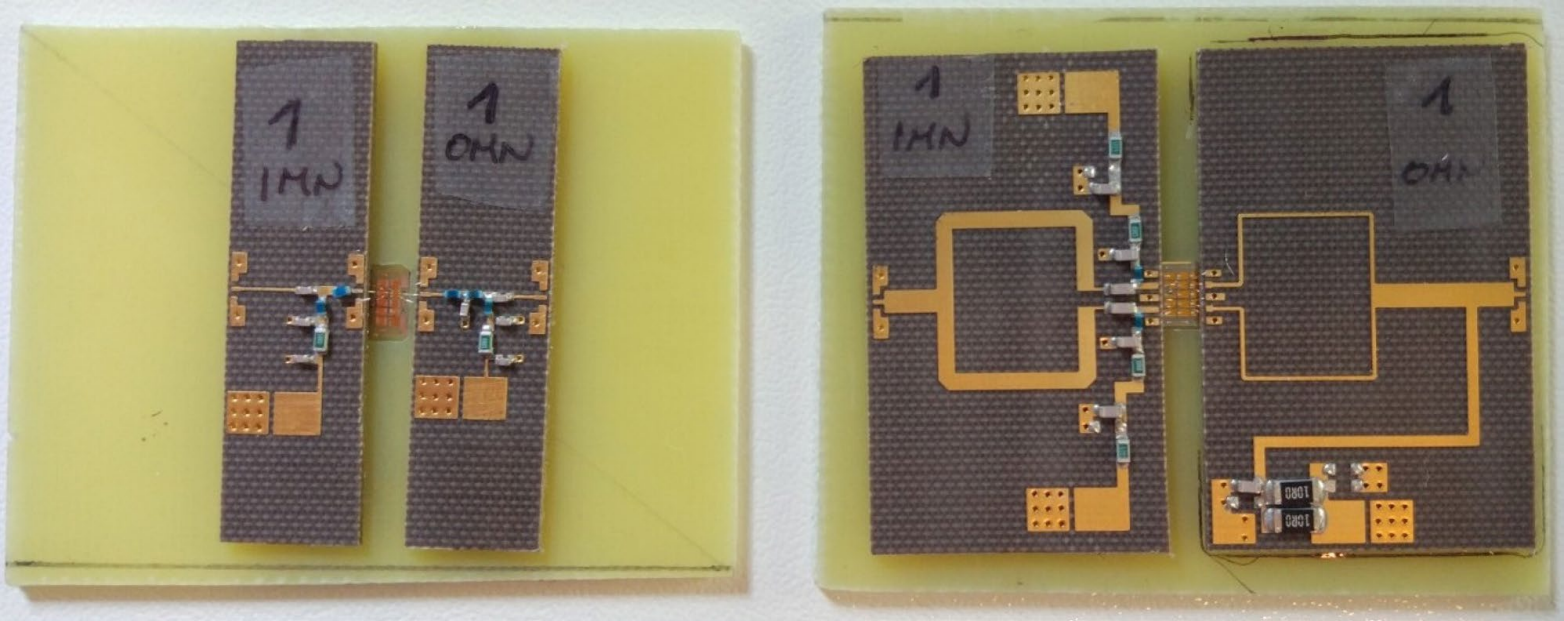
Photographs of the two amplifier stages. Left: low-noise stage. Right: high-power stage.

Then, after selecting the active devices, both the diamond samples and the matching network pairs were glued onto rigid supports, namely, FR-4 with 1.6 mm thickness. The diamond samples were placed inside ad hoc 600 µm recesses, so as to allow placement of the PCSs on top of the samples themselves, close to the selected active devices. Finally, aluminum microwires were soldered to bond the transistors and the matching networks, as previously detailed. The two test vechicles realized in this work are shown in Fig. 5. In more detail, one 2 × 75 µm MISFET was employed in the low-noise stage and two 2 × 150 µm MISFETs were used in the high-power stage. Further details are given below in the dedicated sections.

For the purposes of the current project, given the low power densities to be handled, no specific measures were taken to dissipate heat. Notice, however, that the heat produced by the transistors can spread out through the large diamond samples (4 mm × 4 mm), since the devices are not diced. Measured output power levels of the fabricated amplifiers agree well with those of the bare devices, showing that detrimental effects due to temperature increase did not take place.

S-parameter measurements were performed on the realized stages by means of the LRRM cal kit, in addition to other characterizations. Specific details relevant to the LNA and HPA designs and measurement campaign are given in the following subsections.

## Results

### Low-noise stage

The target performance of the low-noise stage was lowest possible noise figure, gain higher than 6 dB, input and output return losses better than 10 dB. The design band was tentatively 2–3 GHz, and at least half of it. As to the noise performance, a goal of 7.5 dB was set based on the 6–7 dB values of *NF*_*min*_ at 3 GHz predicted by the model of the bare device. However, the comparative distance of the optimum noise match from the origin of the Smith chart represents a serious obstacle to reaching this noise level.

The final design comprises a 3-element IMN and a 5-element OMN (excluding DC feeds and decoupling elements), connected to a 2 × 75 µm FET nominally operated at *V*_*DS*_ = − 10 V, *I*_*D*_/*W* = − 175 mA/mm. EM simulation of the matching networks, in conjunction with a compact model of the bonding wires ^23^ and the equivalent-circuit model of the active device, foresees a noise figure from about 6 dB to about 8 dB across the whole S-band and a transducer gain from 9 to 7 dB. Also, the targets on return loss are achieved in simulations.

**Figure 6:**
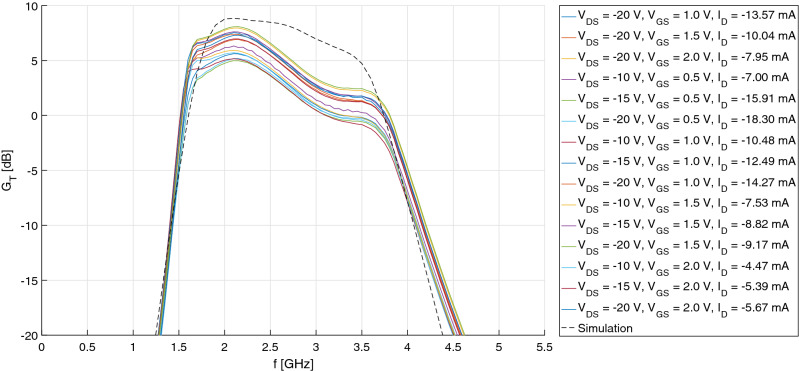
Comparison of the LNA’s transducer gain, measured at different operating points, and simulation.

**Figure 7:**
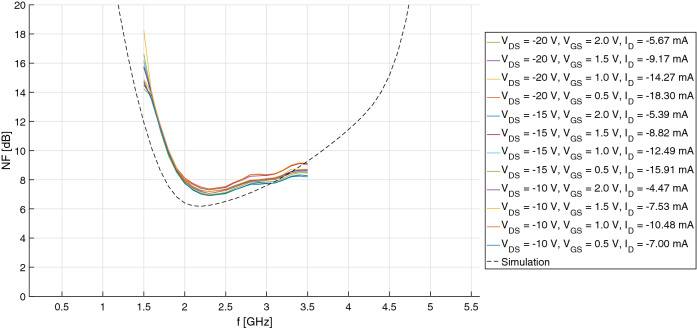
Comparison of the LNA’s noise figure, measured at different operating points, and simulation.

Unfortunately, however, scattering measurements revealed an output return loss much worse than expected (~ 4 dB), which impacted negatively on transducer gain in the upper half of the S-band, as shown in Fig. 6. At least, the IMN fulfilled the 10-dB target on return loss and, more importantly, yielded a measured noise figure comparable to simulations, comprised between 7 and 8 dB: see Fig. 7. This is because the OMN of the single-stage amplifier, being basically reactive, has little effect on output available powers (both signal and noise), and therefore on available gain and noise figure.

**Figure 8:**
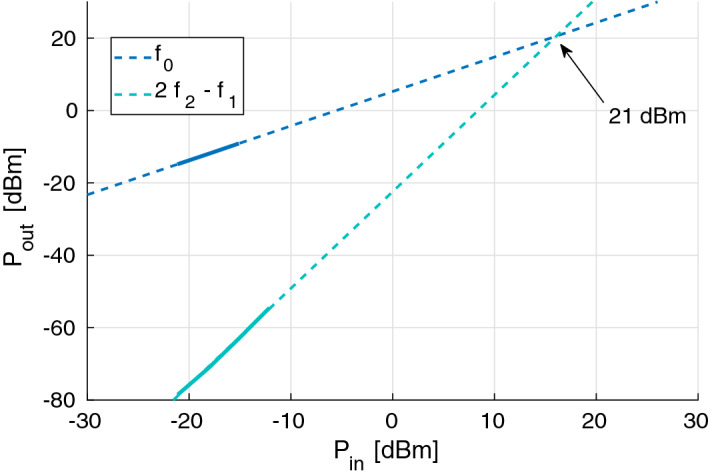
Characterization of the LNA’s third-order intercept point.

Finally, the linearity of the stage was evaluated by means of a 2-tone test, as shown in Fig. 8. The resulting output intercept point is about 21 dBm at 2 GHz.

### High-power stage

The power-oriented stage was designed based on the load-pull and power compression measurements performed during the characterization phase of the ReMiDA project. In order to increase the output power to about 20 dBm, two active devices were combined of geometry 2 × 150 µm and nominal operating point *V*_*DS*_ = − 10 V, *I*_*D*_/*W* = − 200 mA/mm. As to the matching networks and microwires, the same EM simulator and compact models were adopted, respectively, as for the LNA. This time, EM simulations were more largely exploited, due to the distributed nature of the matching networks, which basically consist of a microstrip splitter and combiner. Whereas the output combiner delivers the same drain voltage to both transistors, the input splitter accommodates two separate bias paths for possible tuning. The output combiner also acts as a pair of quarter-wave transformers, mapping the output 50-Ω impedance to the optimum power termination: this was achieved with relative ease, by exploiting the fact that the optimum load is approximately a high resistance, with negligible reactive part.

**Figure 9:**
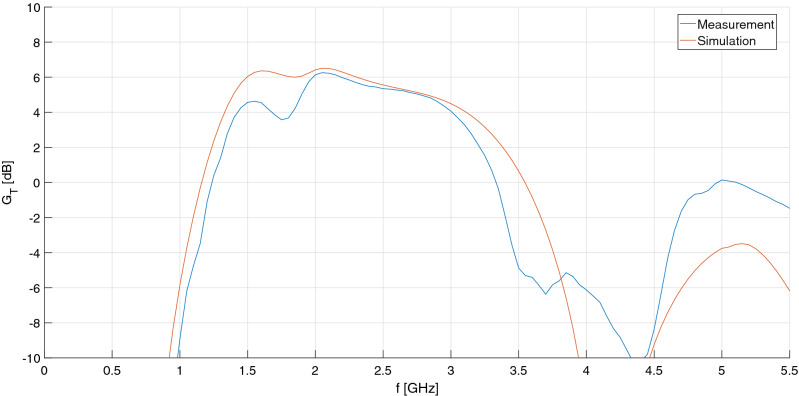
Comparison of the HPA’s measured transducer gain and simulation.

**Figure 10:**
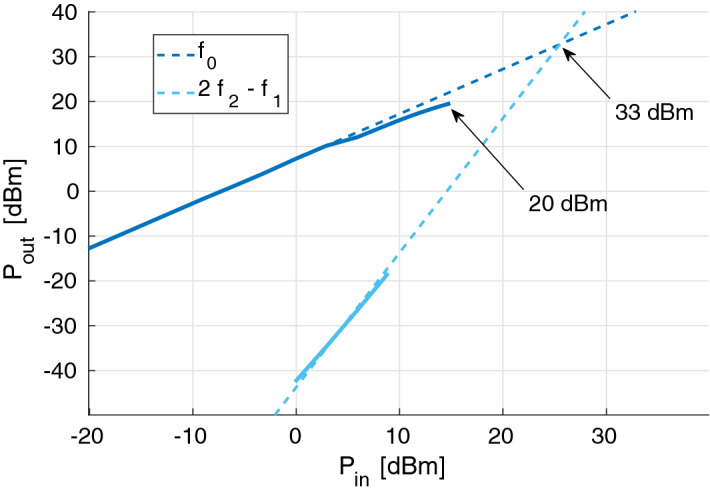
Characterization of the HPA’s compression and third-order intercept point.

The simulated and measured transducer gain of the HPA are compared in Fig. 9: these are in good agreement and amount to about 5 or 6 dB, whereas the measured input and output return losses are again worse than simulated (6 dB and 4 dB, respectively). The measured 1-dB compression point of the HPA is 20 dBm and the third-order intercept point is 33 dBm, both referred to the output section: see Fig. 10.

### Cascade

**Figure 11:**
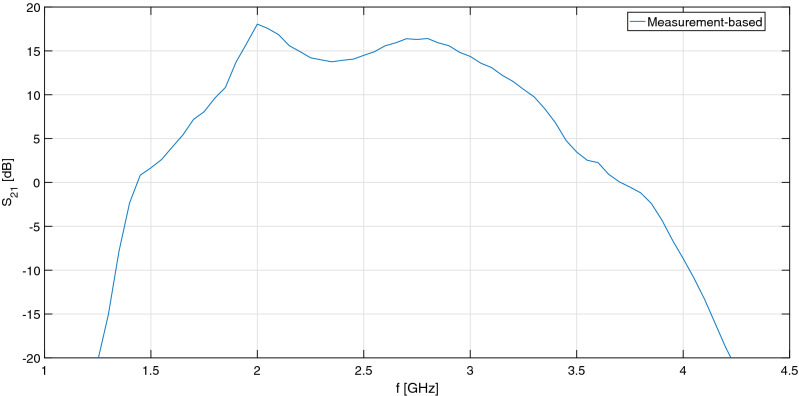
Transducer gain of the LNA-HPA cascade.

Based on the vector-corrected small-signal measurements of the two stages, the scattering performance of the LNA-HPA cascade can be predicted as well. The resulting two-stage amplifier exhibit a nominal transducer gain of 15 dB in the 2–3 GHz band, although with a ripple of ± 1.2 dB, as shown in Fig. 11. The input and output return losses are better than 5 dB and 4 dB, respectively.

## Discussion

For the first time, hybrid amplifiers based on H-diamond FET and operating in S-band are reported. The activities show that the technology still suffers from nonnegligible variability among different realizations, so that the effectiveness of the matching networks can be negatively impacted. Nevertheless, measured performance demonstrates the suitability of H-diamond FETs to microwave applications. The natural evolution of these activities includes the optimization of the technology (stabilization, down-scaling of the gate length) so as to achieve better performance at higher frequencies.

## Data Availability

The Authors make available to readers the collected data and the followed procedures.
